# Molecular pathology and cystogenic propensity of the ADPKD Taiwan founder variant

**DOI:** 10.1172/jci.insight.191419

**Published:** 2025-11-10

**Authors:** Louise F. Kimura, Orhi Esarte Palomero, Megan Larmore, Paul G. DeCaen, Thuy N. Vien

**Affiliations:** 1 Department of Pharmacology, Feinberg School of Medicine, and; 2 Chemistry of Life Processes Institute, Northwestern University, Evanston, Illinois, USA.

**Keywords:** Cell biology, Nephrology, Genetic diseases, Ion channels, Structural biology

## Abstract

Renal polycystins (PKD1, PKD2) are ion channel–forming subunits that traffic to principal cell primary cilia. Variants in these proteins cause approximately 95% of autosomal dominant polycystic kidney disease (ADPKD), a common, lethal genetic disorder that lacks effective drug treatments. We assessed the mechanistic impact and pathogenic propensity of 2 disease-associated PKD2 truncating variants, R803X and R654X. Worldwide, hundreds of individuals with ADPKD harbor these germline mutations, including the R803X founder variant first identified within the patient population of Taiwan. Our biochemical, electrophysiological, and super-resolution imaging analyses demonstrated that the pore-truncating R654X variant abolished channel assembly and ciliary trafficking, whereas the R803X variant retained partial cilia trafficking and channel function. To assess disease impact, we generated transgenic mice with analogous truncation mutations. Homozygous mutants were embryonic lethal, whereas heterozygous mice expressing both variant and conditional *Pkd2* repression alleles developed pronounced renal cysts. Cyst progression was slower in mice carrying the equivalent Taiwan mutation, reflecting the milder clinical course observed in patients. These findings revealed that the degree of impaired PKD2 channel trafficking to primary cilia correlated with cystic disease severity, providing insight into variant-specific ADPKD pathogenesis and newly developed animal models expressing clinically relevant variants for therapeutic testing.

## Abstract 2

Hundreds ADPKD patients harbor PKD2 gene truncating mutations, such as the Tawain founder variant, which confer variable cystogenic propensity driven by differences in molecular dysfunction and primary cilia localization.

## Graphical abstract

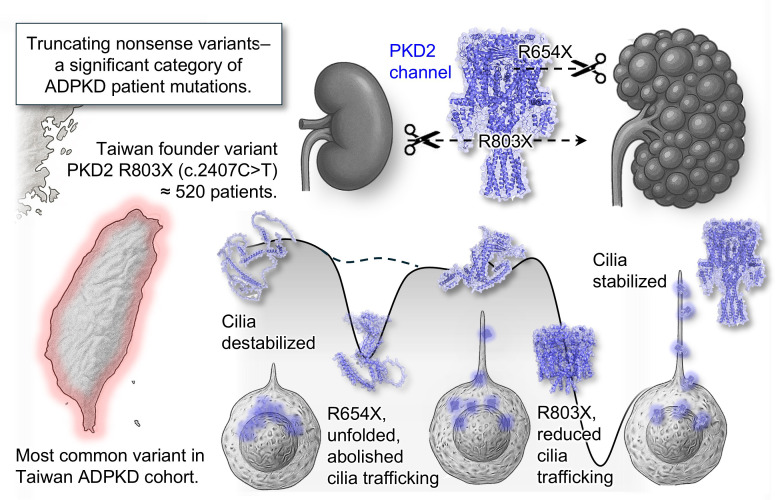

## Introduction

**Figure 1:**
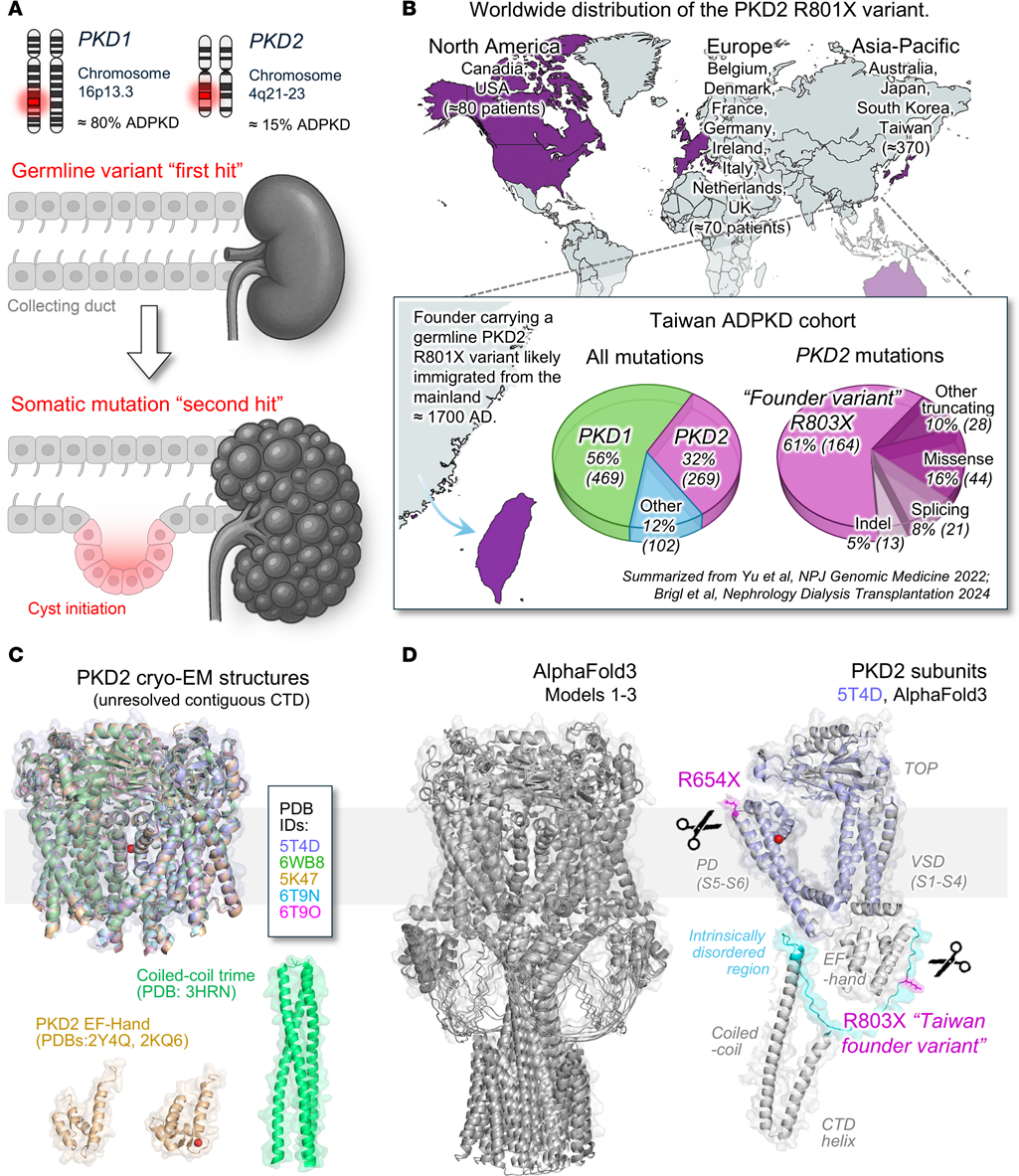
Two-hit hypothesis of ADPKD cyst initiation and the worldwide distribution of the Taiwan founder variant. (**A**) Diagram depicting the germline polycystin gene alleles impacted in 95% of ADPKD clinical cases and the acquisition of a second somatic mutation that drives ADPKD progression. Note, ADPKD does not progress until a second acquired mutation impacts either PKD1 or PKD2 alleles. (**B**) Geographic distribution of the PKD2 R801X truncation variant based on patient sequencing analysis from 25 institutions and 17 countries (29). Expanded view: Published analysis of a cohort of ADPKD patients in Taiwan identifying the PKD2 R801X founder variant (30). Note the high frequency of PKD2 germline mutations (32%) within the Taiwanese patient population compared with that observed globally (~15%). Map images were created with MapChart (https://www.mapchart.net/) under a Creative Commons Attribution–Share Alike 4.0 International License. (**C**) Top: Previously determined PKD2 channel core structures solved by cryo-EM (24, 40, 78, 79). Bottom: The isolated C-terminal EF hand and coiled-coil motif structures solved by NMR and x-ray crystallography, respectively (41, 42, 49). (**D**) Left: Assembled homotetrameric PKD2 channel AlphaFold3 models. Right: A PKD2 subunit modeled using AlphaFold3 identifying the structural location of R654 and R803 variant sites, and integral structural motifs.

Autosomal dominant polycystic kidney disease (ADPKD) is a common monogenetic condition characterized by progressive development of nephrotic cysts that precipitates lethal kidney failure (1). ADPKD impacts more than 12 million individuals worldwide, for whom there is no drug cure. Human variants in renal polycystin ion channel subunits (PKD1 and PKD2) cause the majority of ADPKD (Supplemental Figure 1, A and B) (2, 3). Approximately 80% of patients with ADPKD carry one germline variant in PKD1, and approximately 15% carry a variant impacting the PKD2 gene (4). Results from patient samples and animal models suggest cyst initiation begins when kidney tubule cells acquire a somatic mutation (“second hit”) in either PKD1 or PKD2 alleles (Figure 1A) (5, 6). Thus, ADPKD has a recessive mechanism at the cellular level, which explains the accumulation of renal cysts and typical midlife onset of kidney failure during disease progression (3, 7). Although correlates of genotype to renal function from a limited subset of the ADPKD patient population has been previously reported, our understanding of the cystogenic propensity of the types (missense, nonsense) and the genomic locus of heritable polycystin variants is speculative (8). Beyond the kidney, polycystins have vital roles in the development and maintenance of vital organ systems including the cardiovascular system. Biallelic loss of either PKD1 or PKD2 expression in mice causes embryonic lethality due to cardiac, hepatic, and renal defects (9–11). Various mouse models with hypomorphic expression or conditional loss of polycystin alleles faithfully recapitulate ADPKD phenotypes (12–16). While transgenic mice expressing human disease–associated variants have been reported for PKD1 (17), analogous PKD2 mutations in mouse models are untested. These questions form the premise of our current study, which aims at defining the molecular pathology and cystogenic propensity of PKD2 variants found in the ADPKD population.

ADPKD is a ciliopathic disease, as renal polycystins normally traffic to the primary cilium — a solitary microtubule-based organelle that projects from the apical surface of renal principal cells. Here, homomeric (PKD2) polycystins form voltage-gated and Ca^2+^-modulated channels and are readily measured from primary cilia membranes using submicron-diameter electrodes (18–22). This technique, along with super-resolution methods, was used to determine defective homomeric PKD2 gating properties induced by pathogenic missense variants in the channel’s external TOP and pore helix domains (20, 23). Renal polycystins can also form heteromeric complexes composed of PKD1 and PKD2. However, their functional properties have only been demonstrated under artificial conditions, such as introducing engineered cleavages in PKD2, adding non-native localization signals, or introducing unnatural gain-of-function mutations (e.g., F604P) in PKD2 (Supplemental Figure 1, A and B; supplemental material available online with this article; https://doi.org/10.1172/jci.insight.191419DS1) (18, 19, 24–26). Given their tenability to cilia electrophysiology, we will primarily consider the impact of PKD2 variants within the context of homomeric channels.

Germline truncating variants (nonsense, frameshift) in the PKD2 C-terminus are prevalent in the worldwide ADPKD clinical population (4, 27–29). Within this group, the R803X (p.Arg803*, c.2407C>T) truncating variant is reported in approximately 520 patients in Asia, Australia, Europe, and North America, and was identified as the founder variant of the Taiwanese ADPKD cohort (Figure 1B) (30–32). Haplotype analyses suggest that this variant began propagating within the Taiwanese population around 300 years ago and was likely introduced during the late Ming or early Qing dynasty. Today, the R803X founder variant is present in approximately 32% of the Taiwanese ADPKD patient population, where it is the single most prevalent mutation within this group (Figure 1B) (30). For comparative analysis in this study, we included a more extensive PKD2 truncation variant, R654X (pArg654*, c.1960C>T), which cleaves PKD2 at the channel pore (Figure 1B and Supplemental Figures 1 and 2). This nonsense mutation was identified from ADPKD patients of 9 unrelated families that reside in North America, Europe, and Asia but is not found in geographic concentration (4, 33, 34). While specific longitudinal data on PKD2 truncating variants are limited, PKD2 mutations in general are associated with milder ADPKD, where midlife onset of end-stage renal disease is delayed 10–15 years compared with patients who carry PKD1 mutations (35, 36). However, it is important to note that even among patients with PKD2 mutations, there can be variability in disease severity (36). Ongoing research, including the Taiwan PKD Registry, aims to provide more detailed genotype-phenotype correlations for PKD2 truncating variants and other disease-causing mutations (37). Given the clear clinical importance, we set out to determine the mechanistic impacts of R654X and R803X variants on PKD2 channel trafficking and function in the primary cilia membrane. We then assessed the allelic contribution of these human variants to renal cystogenic propensity by generating 2 new mouse strains that harbor analogous mutations in the PKD2 gene. The in vitro and in vivo findings are correlated to determine how levels of impaired channel biosynthesis (assembly, cilia trafficking) and function confer the onset of ADPKD pathogenicity.

## Results

### Structural impact of the R803X Taiwan founder and R654X pore truncation mutations.

We begin our study by examining which structural motifs would be lost by the R654X and R803X truncating variants, and their anticipated functional effects based on previous work. As described in the Introduction, these 2 germline variants are frequently found within the ADPKD population, but at least 37 other C-terminal truncating mutations are also associated with disease pathology (Supplemental Figure 2) (27, 28). While the high-resolution cryo-EM structures of PKD2 homomeric channels have provided a molecular template of their transmembrane core structure, the C-terminal domains (CTDs) of contiguous channels are unresolved (Figure 1C and Supplemental Figure 1, A and B) (24, 38–40). Nonetheless, the structures of isolated fragments of the C-terminus, including the Ca^2+^-binding EF hand (residues 761–795) and trimers of the coiled-coil motif (residues 836–893), have been determined by crystallographic and nuclear magnetic resonance (NMR) methods (41–43). Initial reports proposed Ca^2+^ occupancy of the EF hand was required for channel function in the endoplasmic reticulum (ER) (44, 45). However, subsequent work challenged this view, where mutations that abolished EF hand Ca^2+^ affinity did not alter PKD2 channel function in the primary cilia (46). Importantly, this null hypothesis was further supported by in vivo observations from 2 mouse strains harboring distinct homozygous EF hand mutations (*Pkd2^ΔEF/ΔEF^*, *Pkd2^-X-Z/-X-Z^*) that did not result in disease phenotypes (46). Based on previous biochemical and modeling analysis, the coiled-coil motif of PKD2 provides structural stability essential for the formation of heteromeric complexes with PKD1 (Supplemental Figure 1A) (47–49). However, it is unknown how this element contributes to the homomeric channel assembly, function, and primary cilia processes that are vital for kidney function and embryonic development. From the cryo-EM structures and their alignments, we can see that R654 is located at the loop connecting the second pore helix (PH2) to the transmembrane pore forming helix (S6); thus, the truncating variants would terminate protein translation of the inner pore and downstream structural motifs within the CTD (Supplemental Figure 2). To provide a structural context and location of the R803X Taiwan founder variant, we used AlphaFold3 to generate models to predict the full-length homomeric channel (Figure 1D) (50). Results refolded the PKD2 monomer tetragonal opening for polycystins (TOP) domain, voltage sensor domain (VSD), and pore domain (PD), in close agreement with the cryo-EM structures, from which a symmetric oligomeric channel is assembled. Similarly, the CTD helices of the EF hands and the coiled-coil motif adopt the expected secondary structures and orientations, but the latter did not assemble channel trimers as previously proposed (51), rather they oligomerized as tetramers. In the AlphaFold3 PKD2 models, R803 is found in an unfolded region (residues 761–795) of the CTD between the EF hand and coiled-coil motif, which was recently proposed to form an intrinsically disordered region (IDR) of the channel (Figure 1D and Supplemental Figure 2) (52). IDRs lack defined 3-dimensional structural folds yet are demonstrably required for various channel–binding partner interactions (53–55). Taken together, these results show the R654X truncation would clearly render PKD2 without an ion-conducting pore formed by S6 homotypic intersubunit interactions. This contrasts with the speculative functional consequence of the R803X truncation, which would preserve the channel’s integral transmembrane domains (VSD and PD), but remove the C-terminal IDR and oligomer-stabilizing coiled-coil motif.

### PKD2 truncating variants destabilize channel assembly and disrupt primary cilia trafficking.

**Figure 2:**
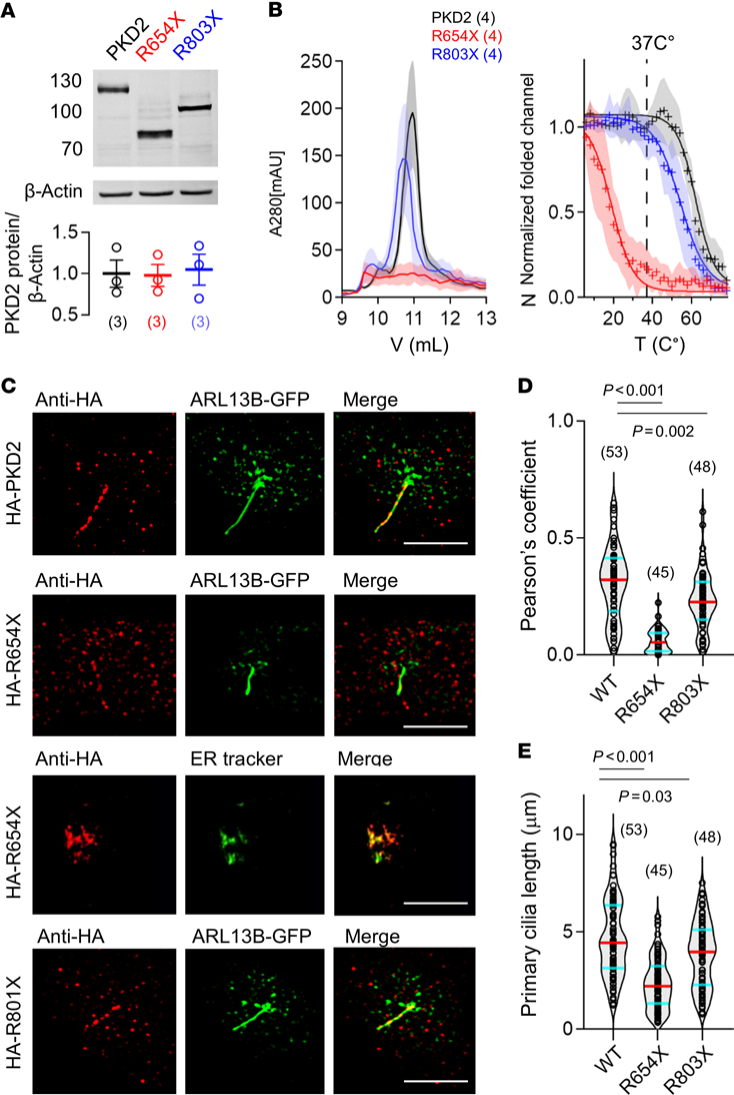
PKD2 truncation variants cause partial to complete loss of homomeric channel assembly and primary cilia trafficking. (**A**) Top: Example Western blot demonstrating differences in the electrophoretic mobility of N-terminally HA-tagged PKD2 and truncation variant channel protein harvested from PKD1^null^:PKD2^null^ HEK293 cells. Bottom: Channel protein expression was quantified by anti-HA intensity (1:2000; 26183, Invitrogen) relative to β-actin expression loading control (1:5000; MA5-15739, Invitrogen). (**B**) Left: SEC analysis of protein lysates harvested from PKD1^null^:PKD2^null^ HEK293 cells transiently transfected with plasmids encoding HA-PKD2 and truncation variant channels. Right: FSEC thermal stability analysis of the assembled channel proteins. Results were fit to a sigmoid equation to compare the destabilizing effects of heat on PKD2 protein oligomers. (**C**) Super-resolution structured illumination microscopy (SIM) images of PKD1^null^:PKD2^null^ HEK293 cells stably expressing ARL13B-GFP to visualize the primary cilia. HA-PKD2 and variant channels immunolabeled with anti-HA. Cells expressing the R654X variant were colabeled with an ER tracker (Glipalamide, Invitrogen). Scale bars: 3 μm. (**D**) Cilia channel fluorescence colocalization analysis using the Pearson’s correlation coefficient. (**E**) Comparison of cilia length from the indicated HEK293 cell lines. *P* values indicate 2-tailed Student’s *t* test results (see Methods) comparing WT and variant channels (*P* < 0.05). Sample size (cilia) for each group are indicated in the parenthesis and error bars represent SD.

To first biochemically evaluate the impact of ADPKD-associated R654X and R803X truncation variants heterologously, we expressed N-terminally HA-tagged variant channels (HA-PKD2) in PKD1^null^:PKD2^null^ HEK293 cell lines genetically edited with the CRISPR/Cas9 method (23). Using this expression system, we can evaluate the impact of channel variants in kidney cells without the contribution of endogenous polycystin subunits. PKD2 protein expression was verified from cell lysates using antibodies raised against the HA epitope, where immunogenic bands from the truncation mutants separated with faster electrophoretic mobility (Figure 2A). To evaluate their impact on channel oligomerization and thermal stability, channel proteins in *n*-dodecyl β-D-maltoside (DDM) detergent were affinity purified using size-exclusion chromatography (SEC) at 4**°**C and evaluated over a denaturing temperature gradient, using fluorescence SEC (FSEC) to detect protein tryptophan fluorescence (Figure 2B and Supplemental Figure 3) (56). Full-length PKD2 (WT) and R803X tetramers were purified in abundance but R803X was less stable at temperatures above 37°C. In contrast, only nominal amounts of the R654X oligomeric protein were eluted and the small fluorescence signal was highly unstable when heated. Using structured illumination microscopy (SIM), we first compared WT, R654X, and R803X channel trafficking to the primary cilia using immunofluorescence. As we have reported previously, we then stably expressed the primary cilia reporter C-terminally GFP-tagged ADP ribosylation factor–like GTPase 13B (ARL13B-GFP) in our PKD1^null^:PKD2^null^ HEK293 cell lines (Figure 2C) and transiently transfected PKD2-encoding plasmids (23). Imaging analysis of fixed cells indicated complete abolishment of R654X from primary cilia trafficking and that the misfolded channel protein remained internally localized (Figure 2D). The fraction of the R803X founder variant still trafficked to the cilia, but with reduced localization, as assessed by Pearson’s coefficient analysis with the ciliary ARL13B signal. Cells expressing either truncation variants had statistically (*P* < 0.05) shorter primary cilia compared with WT PKD2, with R654X being the most impacted (Figure 2E). The biochemical results indicate R654X abolishes oligomerization and stability, resulting in loss of channel delivery to primary cilia. This outcome is likely a consequence of complete removal of the CTD and the S6 homotypic interactions that form the channel pore (Figure 1D). By comparison, the R803X Taiwan founder variant only partially destabilizes channel tetramers despite partially missing its IDR and completely removing the oligomerizing coiled-coil motif (Figure 1D) (43, 47, 49). Taken together, these data correlate the rank order variant impacts on channel assembly and organelle trafficking with primary cilia stabilization. Since R803X ciliary trafficking was attenuated but not completely abolished, we hypothesized that this variant channel may still partially function in the organelle membrane.

### PKD2 truncating variants reduce channel availability in the primary cilia.

**Figure 3:**
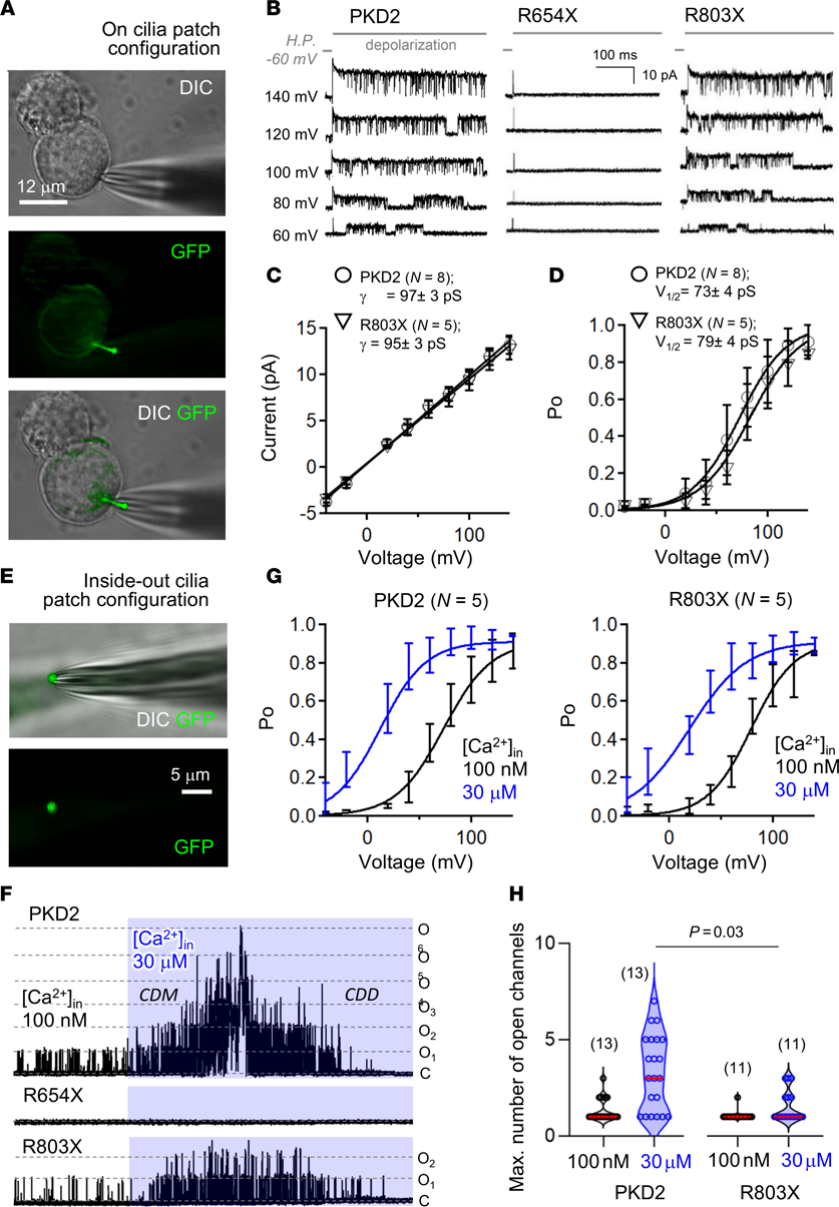
Founder mutation R803X reduces the number of available primary cilia channels without impacting their function. (**A**) Left: Images of a voltage-clamped primary cilia in the on-cilia configuration. Primary cilium of PKD1^null^:PKD2^null^ HEK293 cells expressing PKD2 channels are illuminated by ARL13B-GFP under 475 nm laser excitation. Scale bar: 12 μm. (**B**) Example single-channel recordings from cilia membranes activated by depolarizing voltage steps. Note, no open-channel events were observed in cilia patch recordings from cells expressing R654X channels. (**C**) Unitary conductance was estimated from the slope (γ) of a linear fit of the single-channel current magnitudes. (**D**) Open probability (Po)–voltage relationships were fit to a Boltzmann equation to estimate half-activation voltage (V_1/2_). (**E**) Left: Images of a voltage-clamped primary cilia membrane in the inside-out configuration. Note that the cilium is separated from the cell body, exposing the inner side of the membrane where internal calcium concentration ([Ca^2+^]_in_) was modulated through bath conditions, as previously described (46). (**F**) Exemplar continuous inside-out PKD2 single-channel recording where initial stimulatory calcium-dependent modulation (CDM) and subsequent desensitization (CDM) effects are initiated by increasing [Ca^2+^]_in_ from 100 nM to 30 μM. Note: Fewer simultaneous channel openings are stimulated from R803X cilia membrane patch (2 channels, O_2_) compared with WT PKD2 (6 channels, O_6_). Scale bar: 5 μm. (**G**) CDM of WT and R803X single channels as demonstrated by robust shifts in the V_1/2_. (**H**) Violin plots of the maximum number of simultaneous open-channel events before and after calcium stimulation. The number of cilia records for each data set is indicated within the parenthesis. *P* values indicate results from 2-tailed, unpaired Student’s *t* tests comparing WT and variant channel data sets.

Primary cilia compartments are highly insulated from the cell body, where their contributions to plasma membrane currents are nominal when measured from native collecting duct cells or when the channel subunits are overexpressed (19). Using our previously reported technique, primary cilia were visualized from PKD1^null^:PKD2^null^ HEK293 by stably expressing the ARL13B-GFP reporter (20, 23). High-resistance microelectrodes were used to measure single-channel open events in the “on-cilia patch” configuration (Figure 3, A and B) (20, 23, 57). The half-maximal voltage (V_1/2_) required to open the PKD2 channels was determined by fitting the open probability (Po)–voltage relationship to a Boltzmann equation, where the impacts of mutations on channel gating properties can be assessed (18, 20, 46, 58). Here, R803X founder variant did not affect the unitary conductance or V_1/2_ when compared to the WT PKD2, suggesting that the truncated channel’s ion permeation and gating are unaltered (Figure 3, C and D, and Supplemental Table 1). We empirically noted that far fewer stochastic single-channel events were observed from cilia patches expressing the Taiwan founder variant than WT channels, and none recorded from the R654X variant (Figure 3B). To capture this difference in more quantitative terms and to further probe the biophysical regulation of the variants, we established “inside-out cilia patch” configurations and adjusted intraciliary Ca^2+^ using the superfusate to initiate Ca^2+^-dependent modulation (CDM) and subsequent Ca^2+^-dependent desensitization (CDD) (Figure 3, E and F) (46, 59, 60). Like WT channels, the V_1/2_ of the Taiwan founder variant shifted to negative potentials when internal Ca^2+^ was elevated, which is consistent with a normal CDM response (Figure 3F and Supplemental Table 1). In separate experiments, we held cilia membrane potentials to +40 mV and measured the number of simultaneous open-channel events after elevating intraciliary Ca^2+^ and their subsequent desensitization (Figure 3, G and H). Both WT and R803X channels were responsive, suggesting both channels can undergo CDM and CDD, and that structural motifs downstream of R803 are not involved in either mechanism (Figure 1D and Supplemental Figure 1). However, the mean maximum number of simultaneous open-channel events activated by 30 μM intraciliary Ca^2+^ was reduced from 3 channels (WT) to 1 (R803X), reflecting the partial trafficking defect of this variant (Figure 3, F and G). As observed in the on-cilia patch configuration, no R654X single-channel events were observed in cilia membranes’ inside-out patches, even after Ca^2+^ stimulation (Figure 3, A and F). These results reinforce our conclusion that the R654X variant causes complete channel retention by abolishing oligomeric destabilization, which excludes its functionality in the cilia membrane. In contrast, the R803X variant causes partial loss of channel oligomerization and cilia trafficking defects, resulting in reduced numbers of activatable channels in the cilia membrane. Based on these results, we hypothesized that degree of defective channel biosynthesis, cilia trafficking, and function induced by the truncation variants should track with in vivo pathology, which we assess in the next section.

### Homozygous expression of PKD2 truncation variant alleles cause embryonic lethality.

**Figure 4:**
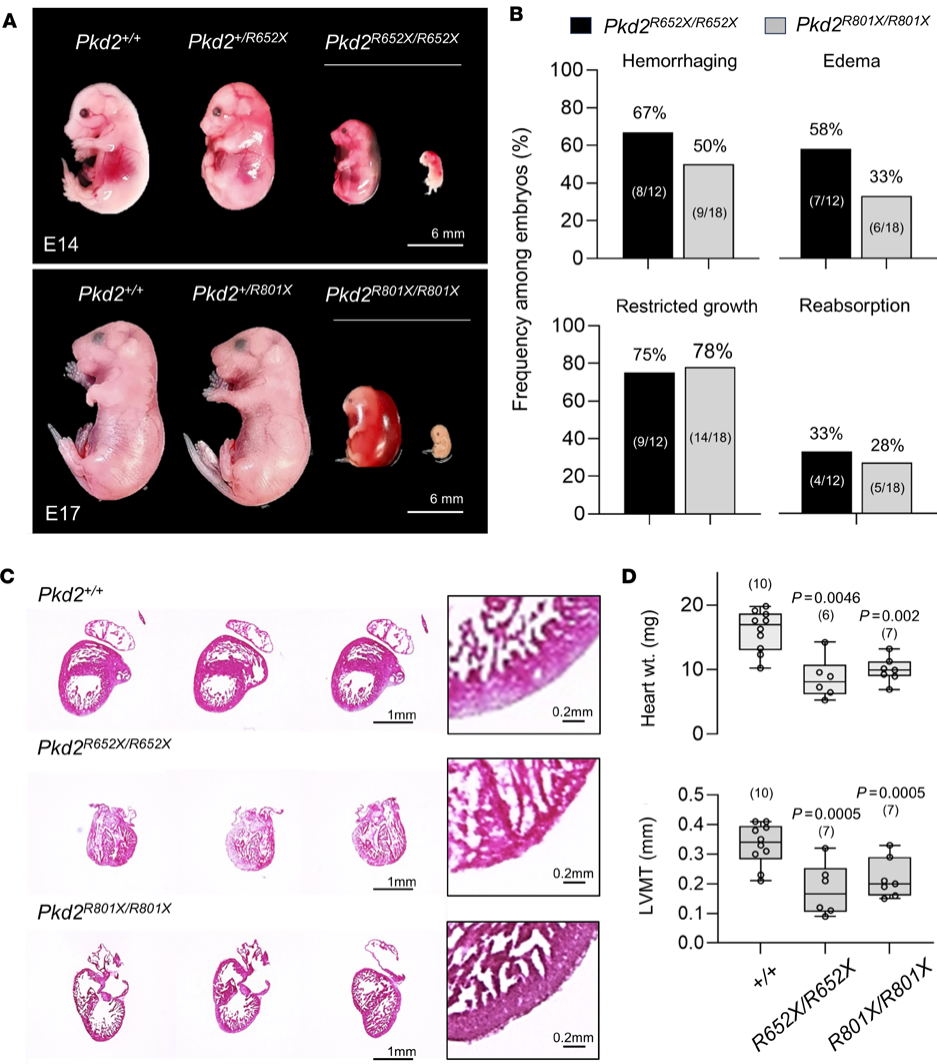
Mice expressing homozygous PKD2 truncation variant alleles are embryonic lethal with cardiac septal defects. (**A**) E14 and E17 embryos expressing human variant truncation alleles *Pkd2^R652X^* and *Pkd2^R801X^*. Note: Homozygous variant embryos are observed in stages of in utero reabsorption, undersized with edema and/or hemorrhaging. Scale bars: 6 mm. (**B**) Bar graphs reporting the percentage and number of embryos with edema, hemorrhage, undersized, and in nonviable stages of in utero reabsorption. (**C**) H&E-stained cardiac sections from E14 mice with isolated hearts are shown in the inset images. Note the underdeveloped heart size and reduced left ventricular myocardial thickness (LVMT) in the homozygous variant mice, as shown in the expanded magnification window. Scale bars: 1 mm and 0.2 mm (high magnification). (**D**) Box-and-whisker plots of heart weights (top) and LVMT per genotype. The sample size is indicated in the parentheses. Shown are the mean (line within box), SEM (box bounds), and SD (whiskers). *P* values resulting from a 1-way ANOVA statistical analysis are shown above the plots.

From our in vitro results, ADPKD C-terminal truncation variants have ranging effects on PKD2 — from partial to complete loss of channel assembly and primary cilia trafficking. Using the CRIPSR/Cas9 gene-editing method, we generated *Pkd2^+/R652X^* and *Pkd2^+/R801X^* transgenic mice that express heterozygous germline mutations equivalent to clinically relevant human variants R654X (pore truncation) and R803X (Taiwan founder), respectively (Figure 4A and Supplemental Figure 2). Genotypic analysis of the outbred offspring produced *Pkd2^+/+^* (19 out of 89), heterozygous *Pkd2^+/R652X^* (33 out of 89), and *Pkd2^+/R801X^* (37 out of 89) progeny. However, no homozygous (*Pkd2^R652X/R652X^* and *Pkd2^R801X/R801X^*) truncation mutant mice were detected in the litters of either strain (0 out of 89). Timed pregnancy trials captured homozygous truncation variant lethality between E14 and E17, where the genetic identity of embryos in stages of reabsorption was confirmed by sequence analysis. *Pkd2^R652X/R652X^* and *Pkd2^R801X/R801X^* embryos frequently exhibited whole-body edema, polyhydramnios, and/or hemorrhaging (Figure 4B). Additional histological examination identified underdeveloped (smaller) hearts with thinner left ventricular myocardium, features that were also observed in *Pkd2*-null and polycystin gene hypomorphic mouse strains (Figure 4, C and D) (9, 14). Based on this existing precedent, we hypothesize embryonic lethality we observed from the homozygous PKD2 truncation variant strains is caused by cardiovascular defects during development.

**Figure 5:**
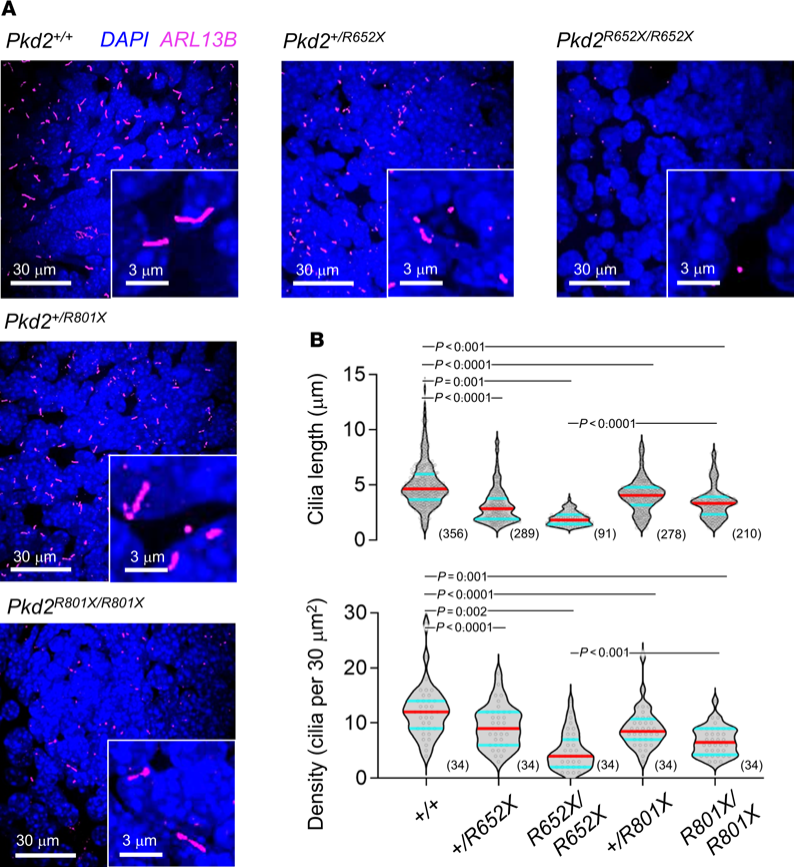
Truncation variants attenuate cilia length in vivo. (**A**) Immunohistochemistry confocal images of embryonic kidney (E14–E16) sections from mice expressing PKD2 truncation alleles. Tissues were fixed in paraformaldehyde, cell nuclei were stained with DAPI, and primary cilia were labeled with anti-ARL13B antibody (Proteintech, 667391-1-Ig). Scale bars: 30 μm (low magnification) and 3 μm (high magnification). (**B**) Violin plots of renal primary cilia length and cilia density. Horizontal red line indicates the average and cyan lines indicate the 25 and 75 percentiles of each data set. The sample size is indicated in the parentheses. *P* values resulting from a 1-way ANOVA statistical analysis of the variants and WT, and 2-tailed Students *t* test between the homozygous genotypes are shown above the plots.

Complete murine renal development occurs at 2–3 weeks after birth; thus, determining nephrology defects embryonically is challenging. Nonetheless, we sought to determine the variants’ impact on embryonic renal primary cilia morphology. Here, embryonic kidneys from littermates expressing variant alleles were harvested, fixed, sectioned, and immunolabeled with anti-ARL13B antibodies to visualize the primary cilia (Figure 5A). We observed statistically fewer and shorter primary cilia from mice expressing either of the variant alleles, but the greatest impact was observed from the *Pkd2^R652X/R652X^* embryonic kidneys (Figure 5B). Thus, these ex vivo findings recapitulate the in vitro observations, where disrupted cilia morphology was observed in HEK293 cell lines expressing the channel variants. Western blot results of embryonic renal tissue did not detect differences in total PKD2 protein from mice expressing homozygous *Pkd2^+/+^* and truncation alleles (Supplemental Figure 4A). However, the biotinylated surface expression of the R652X mutation was abolished, although there was insignificant impact on the equivalent R803X founder variant (Supplemental Figure 4B). These observations support the conclusion from our in vitro analysis, where R654X dramatically reduces protein thermal stability and channel assembly, ultimately eliminating surface expression of the pore truncation variant in vivo. Furthermore, these findings implicate aberrant cilia morphology as a cellular feature of polycystin allelic dysfunction, specifically related to reduced channel assembly and primary cilia trafficking.

### Renal cystogenic propensity of the Taiwan founder and pore truncation variants.

**Figure 6:**
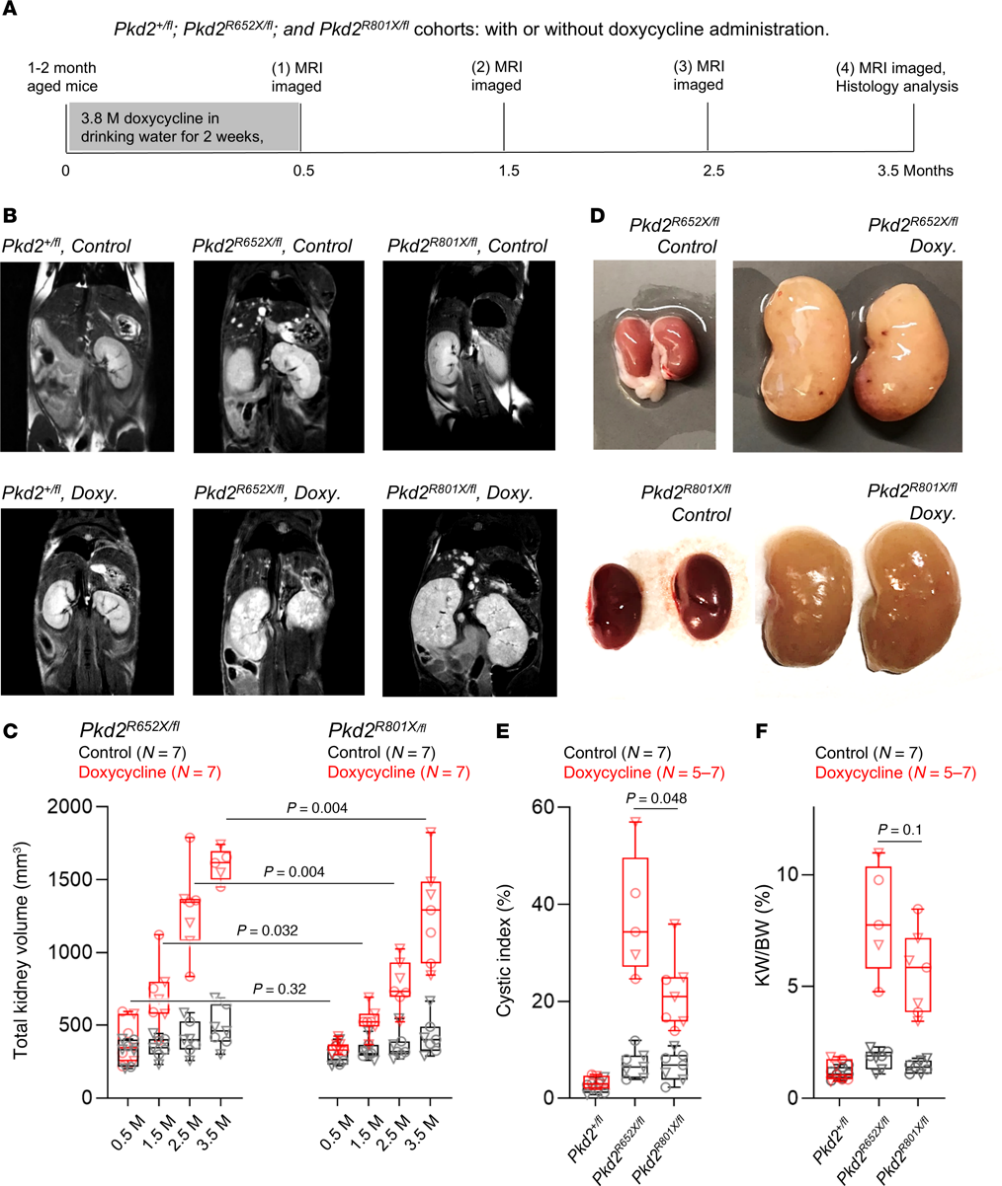
Compound transgenic mice expressing analogous PKD2 truncating variants have different rates of disease progression in vivo. (**A**) Diagram describing the vivo study. Cohorts consisted of *Pkd2^+/fl^* (untreated group *n* = 3 male, 4 female; doxycycline treatment group *n* = 4 male, 4 female); *Pkd2^R652X/fl^* (control group *n* = 4 male, 3 female; doxycycline *n* = 3 male, 4 female) and *Pkd2^R801X/fl^* (untreated group *n* = 3 male, 4 female; doxycycline *n* = 4 male, 3 female) were scanned by MRI monthly. M, months. (**B**) Thoracic cavity MRI of compound transgenic mice expressing a single PKD2 allele under kidney-specific doxycycline repression (*Pkd2^fl/fl^*), and one of the PKD2 truncation mutation’s (*Pkd2^R652X/fl^* and *Pkd2^R801X/fl^*) alleles. Mouse cohorts (control or doxycycline) were treated through drinking water at 1–2 months of age. Example images were take from mice 3.5 months after treatment. (**C**) Onset of PKD from compound transgenic mice expressing truncation variants after monoallelic ablation. Box-and-whisker plots of kidney volumes estimated from monthly MRI analysis. Results from individual male mice are represented by triangles and females represented circles. Welch’s *t* test statistical analysis for doxycycline-treated *Pkd2^R652X/fl^* and *Pkd2^R801X/fl^* cohorts at each month are shown. (**E** and **F**) Box-and-whisker plots of kidney section cystic index and kidney-to-body weight ratio analyzed at the termination of the study. The average for the genotype is indicated by an horizontal line. The number of animals per genotype are indicated within the parentheses. Results from Welch’s *t* test statistical analysis are shown. Box-and-whisker plots show the mean (line within box), SEM (box bounds), and SD (whiskers).

Given the apparent embryonic developmental dependence of at least one fully functional PKD2 allele, we examined the cystogenic propensity of heterozygous *Pkd2^+/R652X^* and *Pkd2^+/R801X^* mice. Histology was performed after 6–7 months of life where hepatic, lateral, and bilateral renal cysts were present in several individuals within the *Pkd2^+/R652X^* cohort, but fewer were observed in *Pkd2^+/R801X^* strain (Supplemental Table 2). The relatively low-penetrant renal phenotype among the heterozygous variant strains is not surprising given that heterozygous null (*Pkd2^+/–^*) mice have low incidence of PKD (9). Thus to further evaluate the cystogenic propensity of truncation variants, we crossed *Pkd2^+/R652X^* and *Pkd2^+/R801X^* mice with the kidney-specific conditional *Pkd2*-floxed repression strain *Pax8^rtTA^; Tet-O-cre; Pkd2^fl/fl^* (abbreviated *Pkd2^fl/fl^*) previously developed by the Somlo laboratory (16). The resulting compound heterozygous mice (*Pkd2^R652X/fl^* and *Pkd2^R801X/fl^*) express the human equivalent truncating mutation on one allele, while the other *Pkd2^fl/fl^* allele is repressible after doxycycline treatment. As expected, *Pkd2^R652X/fl^* and *Pkd2^R801X/fl^* mice were viable, and cohorts to evaluate cystogenesis were established. Conditional *Pkd2* gene inactivation was induced in the cohorts (1–2 month aged mice, *n* = 7) by introducing doxycycline (2 mg/mL or 3.9 mM) into drinking water for 2 weeks (Figure 6A). Control groups were not administered doxycycline for comparative analysis. All cohorts were subjected to monthly thoracic cavity magnetic resonance imaging (MRI) analysis to compare cyst progression over 3.5 months (Figure 6, B and C). MRI results detected progressive enlargement of kidney volumes along with renal and hepatic cyst formation from the drug-treated groups of both mouse strains (Figure 6C). When comparing the MRI results from the doxycycline-treated floxed cohorts, the onset of the disease from the *Pkd2^R652X/fl^* strain was more rapid and pernicious than that observed in *Pkd2^R801X/fl^* mice. Of note, 2 mice from the *Pkd2^R652X/fl^* doxycycline treatment group were humanely euthanized 12–13 weeks into the study due to suffering. Histological analysis performed at the conclusion of the study identified enlarged kidneys with proliferative renal cysts from the *Pkd2^R652X/fl^* and *Pkd2^R801X/fl^* mice (Figure 6, D–F). Disease progression was more extensive in the *Pkd2^R652X/fl^* mice than the *Pkd2^R801X/fl^* mice, as quantified by the kidney weight–to–body weight ratio and cystic index (Figure 6, E and F). These results suggest PKD2 truncating mutations have rank order of pathogenicity, where the R654X pore variant (R652X mouse) is more cystogenic compared with the R803X Taiwan founder variant (R801X mouse). The resulting newly developed ADPKD mouse models are among the first to our knowledge based on clinically relevant PKD2 variants.

## Discussion

In this study, we investigated the mechanistic effects of 2 clinically relevant PKD2 nonsense variants, R803X and R654X, that collectively impact hundreds of patients with ADPKD (20, 30, 61, 62). Computational genetic analyses predict these truncations to be pathogenic (27) and while our results clearly support this classification, their impact on cilia channel activity is ranging despite having a shared molecular mechanism of dysfunction. Our in vitro results demonstrate that R803X causes partial loss of channel assembly and trafficking to the primary cilia membrane, whereas the R654X variant leads to a complete loss of both features. Importantly, these in vitro differences correlate with cystogenic potential in vivo when these mutations are coexpressed with floxed *Pkd2* alleles. Clinical data from the Taiwan PKD Consortium also align with these findings, as individuals harboring the R803X mutation exhibit slower decline in renal function compared with carriers of larger PKD2 gene truncations (20, 30). Previously, we observed no differences in cilia length in cells expressing partial loss-of-function PKD2 TOP domain variants (20); however, those experiments were conducted in PKD2^null^ HEK293 cells only. Therefore, the differing results may be due to the complete absence of endogenous renal PKD1 and PKD2 gene expression in the current model. Importantly, we found that primary cilia shortening correlated with the severity of variant-induced abolishment of channel trafficking, with cells expressing the R654X truncation exhibiting the shortest cilia. The blunted cilia feature was recapitulated in embryonic renal cells harvested from homozygous mice expressing *Pkd2^R652X^* or *Pkd2^R801X^* alleles. These findings suggest that functional polycystin channel expression is critical for ciliogenesis or stabilizing primary cilia morphology.

### Implications of truncation variants on PKD2 ciliary trafficking and channel structural regulation.

The Taiwan founder variant deletes the IDR and coiled-coil motif of PKD2 that results in impaired cilia trafficking. Yet, a small subset of R803X channels still assemble, traffic, and function within the primary cilium. The biotinylated surface expression of PKD2 channels in embryonic renal tissue from homozygous Taiwan founder variant mice (*Pkd2^R801X/R801X^*) remained comparable to WT levels, indicating that overall “mature” trafficking is largely unaffected by the R803X mutation (Supplemental Figure 3, A and B). However, we observe a partial reduction in PKD2 localization within the primary cilium when the R803X channel is overexpressed. These findings suggest the founder mutant specifically impairs ciliary targeting or retention mechanisms. This disparity likely reflects the distinct trafficking pathways and molecular signals required for ciliary versus general plasma membrane localization. The R803X truncation may disrupt critical motifs or protein interactions necessary for engagement with the ciliary transport machinery, such as intraflagellar transport components, thereby reducing channel abundance in the cilium (63). Nonetheless, the channels that do localize to the cilium retain functionality, albeit at reduced levels, consistent with partial preservation of channel assembly and gating (Figures 2 and 3). These findings highlight the selective vulnerability of ciliary trafficking to the R803X mutation, which may contribute to disease pathogenesis through diminished ciliary channel function despite preserved surface expression elsewhere.

The defining functional properties of homomeric PKD2 channels, CDM and CDD, are maintained in R803X variant channels. This suggests that structural motifs responsible for these forms of channel regulation do not involve the IDR and coiled coil. The EF hand in R803X subunits is preserved, but neutralization of Ca^2+^ affinity at this motif neither impacts the channel function nor contributes to cystogenesis in animal models (46). Thus, the structural motifs driving CDM and CDD of PKD2 in the primary cilium are undetermined. By contrast, the R654X truncation removes the S6 transmembrane helices and C-terminal motifs essential for homotypic interactions that form the ion-conducting pore. This results in complete failure of channel assembly and total loss of trafficking to the cilia. The C-terminal-truncating variants differ mechanistically from gating abnormalities seen in some missense variants of the TOP domain (R322Q/W, R325Q/P, and C331S) and reentrant pore helix 1 (F629S and R638C). Mutations in these structural locations typically do not impair cilia localization except for C632R, which also impairs PKD2 biosynthesis and abolishes channel oligomeric assembly (23, 24). Taken together with prior studies, our data indicate ADPKD pathogenesis arises from distinct mechanisms of PKD2 dysfunction, depending on variant location and type.

### Value of ADPKD animal models expressing clinically relevant variants.

Although ADPKD is a dominant monogenic disease, cyst formation is recessive at the cellular level, requiring somatic inactivation of the remaining normal allele (64). Following the functional differences observed in vitro with the R654X and R803X clinical variants, we hypothesized that the corresponding mouse mutations (*Pkd2^R652X^* and *Pkd2^R801X^*) may have different cystogenic potential in vivo. Homozygous mice expressing either mutation result in embryonic lethality. This phenotype is consistent with homozygous *Pkd2*-null mice (*Pkd2^–/–^*) and is likely related to loss of polycystin Ca^2+^ signaling in embryonic nodal cilia that is essential for cardiac development (14, 65–67). To circumvent lethality, we generated compound transgenic mice (*Pkd2^R654X/fl^* and *Pkd2^R801X/fl^*) that express both the truncation variant and conditionally repressed *Pkd2*-floxed alleles. Both strains developed penetrant PKD; however, cyst progression was more severe in *Pkd2^R652X/fl^* mice, reflecting a greater loss of channel assembly, trafficking, and function observed in vitro. Currently, no curative therapies exist for ADPKD, but targeting polycystin function pharmacologically is a promising therapeutic strategy. Based on our findings, direct activation of functional PKD2 R803X variants with agonists or reinstating the channel cilia trafficking through stabilization with molecular chaperones (“correctors”) is a rational approach. Clinical trials are underway for small-molecule correctors that aim to treat ADPKD in patients with a subset of PKD1 gene variants by correcting defective protein folding to restore function (as reviewed in ref. 68), but the efficacy will need to be evaluated against patients with PKD2 variants. While polycystin-gene-knockout mice are available to study disease pathogenesis, these models are less useful in evaluating polycystin-targeted therapeutics, as they completely ablate the channel gene or carry non–clinically related PKD2 mutations (69, 70). Thus, the developed Taiwan founder variant mice (*Pkd2^R801X/fl^*) address the outstanding need for animal models to evaluate polycystin-targeted drug efficacy in vivo. In contrast, this R654X truncation variant removes the pore-forming S6 required for channel assembly and would likely be resistant to the channel-chaperone drug strategy (45). Nonetheless, its analogous conditional mouse strain (*Pkd2^R652X/fl^*) would be most useful in efficacy studies evaluating gene-corrector and gene-replacement therapies (71).

### Limitations of this study.

The findings presented here are mechanistic in terms of variant-induced molecular dysregulation of PKD2 cilia trafficking and channel function. We have described 2 important endpoints — biochemical dysfunction, as it related to human truncating variants, and 2 new ADPKD mouse models based on these clinically relevant mutations. These are among the first demonstrations to our knowledge of an ADPKD animal model based on the expression of clinically relevant PKD2 variants. However, we acknowledge that the scope of the study does not mechanistically link PKD2 mutations to the types of downstream changes in cell signaling driving PKD pathogenesis. We speculate that sequential loss of channel function causes loss of cilia formation, and that abolishing an undefined cilia-specific signal drives cyst progression (12). Future works should utilize these animal models to define how loss of PKD2 and primary cilia compromises calcium, hedgehog, Wnt/β-catenin, and planar cell polarity pathways. PKD2 can form homomeric, voltage-gated, Ca^2+^-modulated channels in primary cilia, but it also oligomerized with PKD1 to form ligand- or cleavage-activated complexes (19). However, previous work has established that functional PKD2 homomeric channels traffic to the primary cilia without the expression of PKD1 (19, 72). Due to the technical challenges of studying low-abundance PKD1 and the clear tractability of PKD2 homomers for functional assays, our study focused on PKD2 variants primarily in the homomeric context. Thus, a limitation of our work is that we did not directly assess how R654X and R803X variants influence the stability or function of PKD1-PKD2 heteromeric channels either in overexpressed or endogenous contexts. Both truncations impact structural motifs (e.g., coiled coil) implicated in PKD1-PKD2 oligomerization (43, 47, 49), which suggests biochemical (thermal and oligomeric stability) and cell trafficking (cilia localization) defects impacting homomeric PKD2 channels would likely affect their heteromeric complexes as well. Future studies addressing this question will benefit from the animal models established here, providing valuable platforms for comprehensive functional and therapeutic investigations.

## Methods

### Sex as a biological variable.

Sex was not considered as a biological variable. In vivo MRI visualization of cyst progression involved both male and female mice at nearly equal numbers, and similar findings are reported for both sexes. Given the small sample size per group, we did not stratify our statistical analyses by sex, as this would have resulted in underpowered and scientifically unreliable conclusions.

### Pkd2^R652X^ and Pkd2^R801X^ mouse treatments.

To characterize their phenotype, *Pkd2^+/fl^* (control group *n* = 3 male, 4 female; doxycycline treatment group *n* = 4 male, 4 female), *Pkd2^R652X/fl^* (control group *n* = 4 male, 3 female; doxycycline *n* = 3 male, 4 female), and *Pkd2^R801X/fl^* (control group *n* = 3 male, 4 female; doxycycline *n* = 4 male, 3 female) were scanned by MRI monthly. Mouse cohorts aged 1–2 months were administered standard drinking water supplemented with sucrose or drinking water supplement with sucrose and doxycycline (2 mg/mL or 3.9 mM) for 2 weeks mice to genetically ablate PKD2 expression (*Pax8rtTA; TetO-cre; Pkd2^fl/fl^*), as described previously (16, 19). MRI imaging was conducted using a Bruker BioSpec (9.4 Tesla). Mice were anesthetized and placed in a chamber containing 3% isoflurane, and their respiration was monitored for the duration of the scan (73).

### Generation of HEK293 PKD1^null^:PKD2^null^ cells lines and the expression PKD2 variants.

Using our previously generated (source Creative Biogene) CRISPR/Cas9-edited HEK293 PKD2^null^ cell line, we introduced nonsense mutations to both PKD1 alleles to create a PKD1^null^:PKD2^null^ cell line (23). HEK293 PKD2^null^ cells were electrotransfected with PKD1 sgRNAs (CACCGCATAGGTGTGGTTGGCAGC and AAACGCTGCCAACCACACCTATGC) with the All-in-one Cas9 plasmid (Addgene). Cells generated from single-cell clones were selected after 4 weeks of expansion under puromycin selection in a 96-well plate. A PKD1^null^:PKD2^null^ clone was verified by extracting the genomic DNA and sequencing for the introduced STOP codons within PKD1 and PKD2 genes using the following primers: PKD1 fwd (CTGATGGCTTAGGCCCCTACTG); PKD1 rev (CCTGGGTCTCGGTAGATGAACG); PKD2 fwd (AGCCTCAGGGCACAGAACAG); and PKD2 rev (CCACACTGCCCTTCATTGGC). PKD1^null^:PKD2^null^ clones stably expressing ARL13B-GFP (VectorBuilder, pLV[Exp]-Puro-CMV) were established after 30 to 90 days or 9–10 rounds of puromycin culture selection (2 μg/mL). Cells were then fluorescence-activated cell sorted (BD FACSMelody) at 5000 to 10,000 counts per minute to enrich for the transgene expression. To generate the WT and variant PKD2 N-terminally HA-tagged variant expression plasmids, the human PKD2 gene was subcloned into mammalian expression vector pRP[Exp]-CMV>HA (VectorBuilder). Missense variants were generated using standard site-directed mutagenesis. Cell lines were cultured in Dulbecco’s modified essential medium (DMEM) supplemented with 10% fetal bovine serum (FBS), 100 U/mL penicillin, and 100 U/mL streptomycin and/or 1 μg/mL puromycin.

### Western blot analysis of overexpressed and endogenous PKD2 channels.

PKD1^null^:PKD2^null^ HEK293 cells expressing WT HA-PKD2 and truncation variants were lysed in buffer containing 100 mM HEPES-NaOH (pH 7.9), 0.9% NaCl, 10 mM EGTA, 5 mM EDTA, 1% Triton X-100, and protease and phosphatase inhibitors. Samples were centrifuged at 15,000*g* for 15 minutes at 4°C. Protein concentration was determined by the Bradford method (74). Aliquots containing 60 μg of tissue or 20 μg of cell extract protein in Laemmli buffer were subjected to polyacrylamide gel electrophoresis (6% SDS-PAGE) with reducing conditions. Proteins were then transferred to a nitrocellulose membrane (Bio-Rad). After blocking with 5% BSA in TBS-T (20 mM Tris-HCl, 150 mM NaCl, and 0.1% Tween 20) for 1 hour, membranes were incubated with anti-HA (1:2000; 26183, Invitrogen) or anti–β-actin (1:5000; MA5-15739, Invitrogen) antibodies overnight at 4°C. After incubation for 2 hours at room temperature with anti-mouse IgG DyLightTM 488 conjugated (1:5000; Invitrogen) diluted in 5% BSA in TBS-T, bands were visualized using iBright Imaging Systems (Thermo Fisher Scientific). Results were analyzed by comparing the mean of normalized band quantification among different genotypes. One-way ANOVA was used for data analysis followed by Holm-Šídák (R801X mutation) or Kruskal-Wallis (R652X mutation) multiple-comparison test. To isolate endogenous PKD2 protein, developing renal tissues from embryos (E14–E17) were dissected and homogenized by sonication in the aforementioned lysis buffer on ice for 30 minutes. Surface biotinylation was performed by incubating cells on ice for 10 minutes with 0.3 mg/mL sulfo-NHS-SS-biotin (21331, Thermo Fisher Scientific) followed by 3 rinses with PBS. Cells were then incubated for 5 minutes with 1 mL of 100 mM NH_4_Cl and 250 μL lysis buffer was added to the cells. The lysed cells were transferred into Eppendorf tubes and centrifuged at 15,000*g* for 30 minutes at 4°C. The supernatant was transferred to a new Eppendorf tube containing 150 μL of 50:50 slurry of streptavidin agarose beads (20359, Thermo Fisher Scientific) and rotated for 1 hour at 4°C. The homogenate was processed for SDS-PAGE as described above. After transfer and 5% BSA blocking in TBS-T for 1 hour, membranes were incubated with anti-PKD2 (1:500; WH0005311M1, Sigma-Aldrich) or anti–β-actin (1:5000; MA5-15739, Invitrogen) antibodies overnight at 4°C, followed by secondary antibody anti-IgG rabbit DyLight 488 (1:5000; 35552, Thermo Fisher Scientific).

### Expression, SEC purification, and FSEC thermal denaturing of PKD2 channels.

PKD2 WT and truncation variants were expressed by transient transfection in Expi293F GnTl– cells (Invitrogen) using an N-terminal Strep-tagged MBP-TEV-HA-PKD2 construct under control of a CMV and β-globin intron promoter. Expi293F GnTl^–/–^ cells were grown at 37°C to 3 × 10^6^ cells/mL density and transfected with PEIMax (Thermo Fisher Scientific) at a DNA/PEIMax ratio of 1:5. Sodium butyrate (10 mM; Sigma-Aldrich) was added 20–22 hours after transfection to boost protein expression. Cells were harvested 72 hours after transfection, washed with PBS, and stored at –80°C until further use. Cell pellets were resuspended in Buffer A (25 mM HEPES pH 7.4, 150 mM NaCl, 1 mM TCEP, 1 mM CaCl_2_, 10% glycerol; Sigma-Aldrich) supplemented with complete protease inhibitors. The cells were homogenized by sonication at 30% power and supplemented with DDM (Sigma-Aldrich) in Buffer A to a final concentration of 2% w/v. Membranes were solubilized by gentle nutation at 4°C for 2 hours and cell debris was removed by centrifugation at 20,000*g* for 30 minutes at 4°C. The cleared lysate was applied to 0.5 mL of equilibrated Strep-Tactin Superflow affinity resin (Qiagen) by gravity flow. The column was washed with 30 CV of Buffer A plus 0.05% DDM/0.005% CHS and eluted in 5 CV of Buffer A supplemented with 5 mM D-desthiobiotin (Sigma-Aldrich). Protein concentration was estimated by A280 UV absorption followed by supplementation with amphipol A8-35 (1:3 w/w; Sigma-Aldrich) and incubation for 1 hour at 4°C. Detergent was removed with 2 batches of SM2 Biobeads (10 mg/0.1 mL; Bio-Rad) by gentle nutation at 4°C, first for 1 hour and then overnight. The maltose binding protein (MBP) solubility tag was cleaved simultaneously with the last batch of Biobeads by the addition of TEV Protease. PKD2 peptide (residues 52–793) reconstituted in A8-35 was polished by SEC in a Superdex 200 10/300 GL column using Buffer B (25 mM HEPES pH 7.4, 150 mM NaCl, 1 mM TCEP, 1 mM CaCl_2_; Sigma-Aldrich). Peak fractions eluting at approximately 9.5 mL after injection were pooled and concentrated with 100-kDa-cutoff centrifugation. FSEC-based thermostability assay was performed as previously described (20). Approximately 10 mg of purified human WT and truncation variants from cell lysates were diluted in Buffer A and incubated at preset temperatures in a thermocycler for 10 minutes. Samples were flash frozen in liquid nitrogen, thawed on ice, and cleared of aggregation by ultracentrifugation (10,000*g*, 30 minutes). Channel protein samples were separated on an analytical size-exclusion column (Superose 6, 5/150 GL) at 0.3 mL/min flow rate and protein tryptophans were detected using an in-line fluorescence detector.

### Production of the Pkd2^R652X^ and Pkd2^R801X^ mouse strains.

Single guide RNAs (sgRNAs) for the *Pkd2* R652X (5′-GCTCACGCTCGGTCAGTTCC-3) and *Pkd2* R801X (5′-CGGAGTCATCCAGGCTTCTG-3) nonsense truncations were designed using the CRISPR Design tool (http://crispr.mit.edu/). To edit the C57BL/6J mouse genome (strain 000664, The Jackson Laboratory), single-stranded oligodeoxynucleotide (ssODN) donor templates for the point mutations were synthesized (IDT). An sgRNA plasmid was constructed and linearized, followed by in vitro transcription of sgRNAs using MEGAshortscript Kit (Invitrogen). The yield and quality of sgRNA were assessed by absorbance ratio and gel electrophoresis (75). Transcribed sgRNAs, Cas9 protein (NEB), and 2 donor ssODN templates were microinjected into zygotes, followed by culture and transfer of blastocysts into the uterus of pseudopregnant ICR female mice (76). Founders were identified by PCR amplification of genomic DNA from tail biopsies, followed by sequencing of the PCR products (75). Genotyping of experimental mice was conducted using allele-specific PCR primers (capitalized) for the R652X mutant (TGAGCGCTTGGCTATCTCCA and acctcatggcttagcgagt), R801X mutant (TCAAGAGCTTGCTGAACGAGC and tgtttaccaaggtcttgggcaagca), and WT alleles (CCAGGAACTGACCGAGCGTGA and tgtttaccaaggtcttgggcaagca).

### Electrophysiology recordings of polycystin channels from primary cilia.

The electrophysiologist was blinded by a third party in the laboratory, whereby test groups were assigned a letter to conceal the genetic identity of the cells being evaluated. The identity of the cells remained unknown by the electrophysiologist until the analysis was complete. Ciliary ion currents were recorded using borosilicate glass electrodes polished to resistances of 14–23 MΩ using the cilium patch method described previously (46). Single-channel currents measured in the inside-out configuration were recorded in symmetrical sodium concentrations. All our solutions were formulated with ultrapure water (Milli-Q IQ 7005 water), which has less than 0.29 ng/L Ca^2+^ ions present. For inside-out patch configurations, the internal solution (bath) contained 120 mM NaMES, 10 mM NaCl and 10 mM HEPES. Ca^2+^ was buffered with 5 mM EGTA, 5 mM glycine, 5 mM *N*,*N*′-1,2-ethanediylbis(oxy-2,1-phenylene)]bis[*N*-(carboxymethyl)-,tetrasodium (Na_4_-BAPTA) and 0.5 mM EDTA. Free Ca^2+^ was calculated using Maxchelator and titration of 1 M CaCl_2_ solution (77); pH was adjusted to 7.3 using NaOH. For on-cilia patch configurations, NaMES was replaced with equimolar KCl to neutralize the cell and cilia membrane potential. Standard external solution (pipette electrode) contained 150 mM NaCl, 10 mM HEPES, 2 mM CaCl_2_; pH 7.4. All solutions were osmotically balanced to 300 (±7) mOsm with D-mannitol. Whole-cell currents used to measure CDD were also recorded in symmetrical sodium concentration, placing the internal recording solution in the pipette electrode and the external recording solution in the bath. Data were collected using an Axopatch 200B patch-clamp amplifier, Digidata 1550B, and pClamp 10 software. Single channel currents were digitized at 50 kHz and low pass filtered at 10 kHz. Intraciliary conditions were controlled using an Octaflow II rapid perfusion system (ALA systems) in which the patched cilia and electrode were held in the perfusate stream. Data were analyzed with Igor Pro 7.00 (Wavemetrics). The PKD2 open probability (Po) current-voltage relationships were fitted to a Boltzmann equation, *f*(*x*)=1/(1 + exp[V – V_1/2_]/*k*), to estimate the half-maximal voltage (V_1/2_) required to open the channels.

### Histology, immunocytochemistry, and structure illumination microscopy.

The kidneys from transgenic mice were fixed in 4% paraformaldehyde (PFA) and immersed in 30% sucrose for 24 hours. Tissues were sectioned on a cryostat, mounted on glass coverslips, and stained with hematoxylin and eosin (H&E). Images of medial sections from both kidneys were analyzed using FIJI (ImageJ, NIH) to identify cystoid foramen. Images were processed as black and white, and reverse-negative. The images were then analyzed using the particle analysis search protocol, in which the lower limit threshold for circular foramen was set to 20 μm to identify cystoid foramen in the tissue samples. Immunocytochemistry of sectioned kidneys of medial sections were fixed with 4% PFA, permeabilized with 0.2% Triton X-100, and blocked with 10% BSA in PBS. PKD1^null^:PKD2^null^ HEK293 cells stably expressing the cilia reporter ARL13B-GFP were transiently transfected with plasmids encoding WT HA-PKD2 and truncation variants channels were fixed with 4% PFA, permeabilized with 0.2% Triton X-100, and blocked with 10% BSA in PBS. Cells and tissue were mounted on glass slides and treated with ProlongGold (Invitrogen). HA-tagged channels were visualized by treated with CoraLite 594–conjugated anti-HA polyclonal antibodies (Proteintech, CL594-51064). ER membranes were visualized using ER-Tracker Red (BODIPY TR Glipalamide, Invitrogen). Confocal images were obtained using an inverted Nikon A1 with a 60× silicon oil immersion, 1.3 N.A. objective. Confocal images were further processed with FIJI ImageJ. Super-resolution images using the SIM method were captured under ×100 magnification using the Nikon Structured Illumination Super-Resolution Microscope with piezo stepping. 3D-SIM images were further processed with Imaris software (Oxford Instruments).

### Statistics.

Statistical methods used to determine significance are described in corresponding figure legends and reported as *P* values. Briefly, electrophysiology and imaging datasets were analyzed (GraphPad or Origen) using 1-way ANOVA, paired 2-tailed Student’s *t* tests (equal sample sizes) or unpaired Student’s *t* tests (unequal sample sizes). Welch’s *t* test statistical analysis was used to assess the in vivo MRI results and ex vivo cyst development.

### Study approval.

Animals were housed at AALAS-certified facilities located at Northwestern University. All animal procedures and protocols were approved by the Institutional Animal Care and Use Committees (IACUCs) at Northwestern University.

### Materials availability.

All mammalian cell expression constructs used in this study are available without restriction upon written request to the corresponding author. The conditional *Pkd2^fl/fl^* mouse strain is available from the Polycystic Kidney Disease Research Resource Consortium (PKD RRC). Mouse strains expressing the heterozygous PKD2 truncating variants alleles (*Pkd2^+/R652X^ and Pkd2^+/R801X^*) are available upon request from the corresponding author or will be made available through the PKD RRC (https://www.pkd-rrc.org/) or another repository within 6 months after publication.

### Data availability.

Values associated with the main manuscript and supplement material, including values for all data points shown in graphs and values behind any reported means, are available in the Supporting Data Values file. Associated raw data reported in this paper are deposited at the Northwestern University ARCH library and are available without restriction (https://doi.org/10.21985/n2-vqcr-ka87).

## Author contributions

LFK and OEP designed experiments, analyzed data, and conducted the functional, biochemical, and localization studies. LFK and OEP contributed equally and were assigned co–first authorship. Author order reflects relative contributions to experimental design and data analysis. ML conducted in silico structural analysis. TNV designed experiments, analyzed data, conducted super-resolution microscopy, in vivo MRI, associated animal husbandry, and subsequent ex vivo histology studies. PGD supervised the study and provided funding. TNV and PGD wrote the manuscript.

## Funding support

Ruth L. Kirschstein NRSA individual postdoctoral fellowship (F32DK137477-01A1 to OEP).

- Northwestern University KUH training grants (U2CDK129917 and TL1DK132769 to OEP).
- National Institute of Diabetes and Digestive and Kidney Diseases (R01 DK123463-01 and R01 DK131118-01 to PGD).
- Sao Paulo Research Foundation (FAPESP-2019/26414-2 to LFK).

## Supplementary Material

Supplemental data

Unedited blot and gel images

Supporting data values

## Version 1 11/10/2025

Electronic publication
