# Supplementary material for: Molecular pathology and cystogenic propensity of the ADPKD Taiwan founder variant
Source: JCI Insight. 2025 Nov 10;10(21):e191419. doi: 10.1172/jci.insight.191419 (PMC12643504; doi:10.1172/jci.insight.191419)
Supplement: Supplemental data [file jciinsight-10-191419-s081.pdf]

SUPPLEMENTAL FIGURES AND TABLES

Molecular pathology and cystogenic propensity of the ADPKD Taiwan founder variant

Authors and affiliations:

Louise F. Kimura[1]*, Orhi Esarte Palomero[1]*, Megan Larmore[1], Paul G. DeCaen[1,2]**, Thuy N. Vien[1]**

1) Department of Pharmacology, Feinberg School of Medicine, Northwestern University, Chicago, Illinois, USA.
2) Chemistry of Life Processes Institute, Northwestern University, Evanston, Illinois, USA

*Authors contributed equally to this work.

**Corresponding authors.

Corresponding authors contact information.

Paul G DeCaen, 320 East Superior, Chicago IL 60601; 206-403-8811; paul.decaen@northwestern.edu

Thuy N. Vien, 320 East Superior, Chicago IL 60601; 206-854-9450; thuy.vien@northwestern.edu

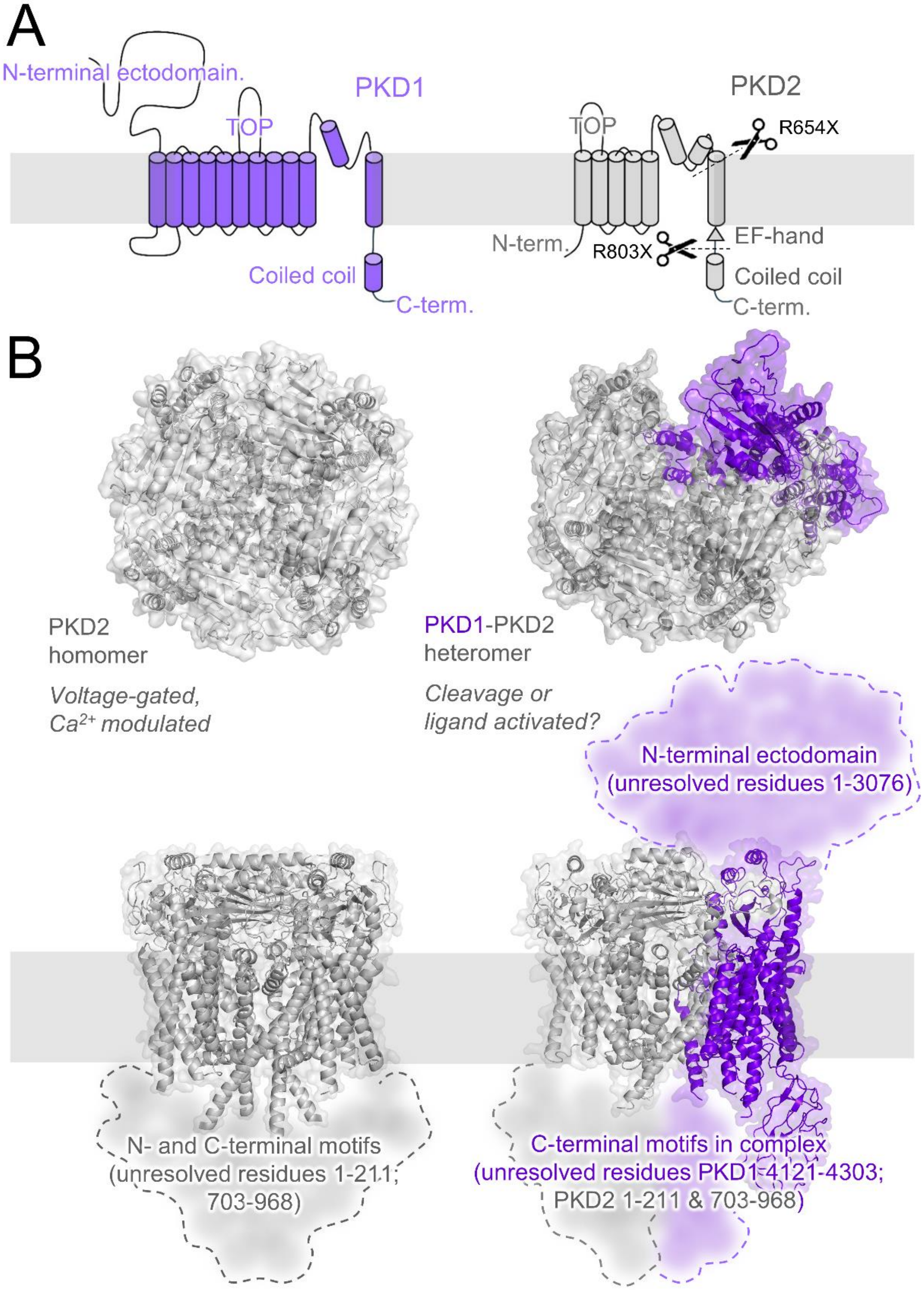

Supplemental Figure 1. PKD2 may form homomeric or heteromeric ion channel complexes. A) toplogy of PKD1 and PKD2 polycytsin channel subunits. B) Cryo-EM structures of voltage-gated, calcium modulated homomeric PKD2 (PDB ID: 5T4D) channel and putative ligand or cleavage activated PKD1-PKD2 (PDB ID: 6A70) channel complex(1, 2). Top row images are external views and bottom row images are transmembrane views of the channnel structures. Note the large portions of the channels which are structurally unresolved.

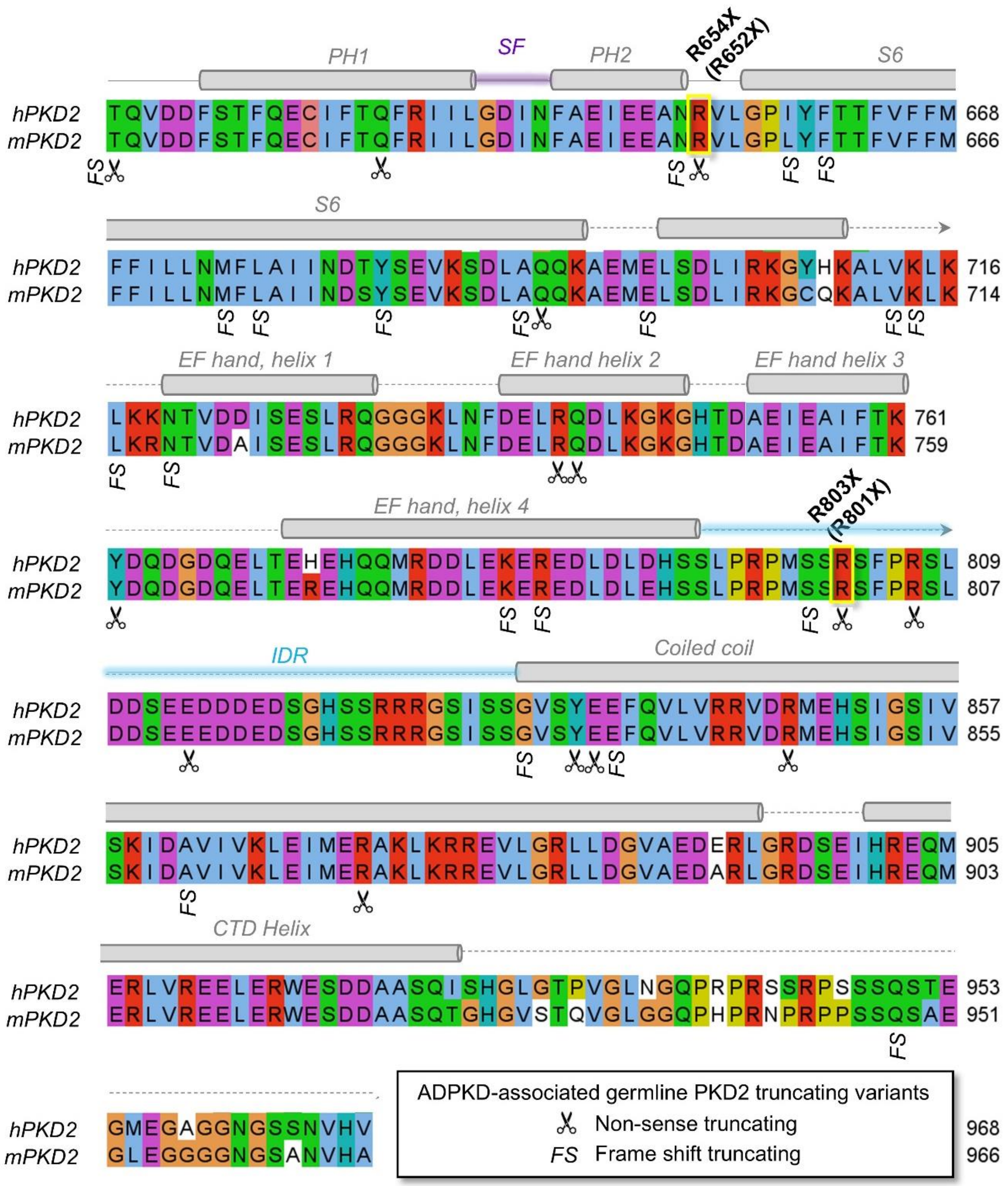

Supplemental Figure 2. Location of ADPKD-associated truncating variants. C-terminal amino acid Clustal-omega aligment of human and mouse PKD2 rendered in JalView applying the default color scheme: Hydrophobic (blue); polar (green); glutamate, aspartate (purple). Special amino acids are designated with their own color: glycine (orange); proline (yellow) and tyrosine or histidine (cyan). The barrels indicate alpha helices found in the PKD2 AlphaFold3 structures and the location of ADPKD-assocated truncating varriants (non-sense, frameshift) are indicated(3).

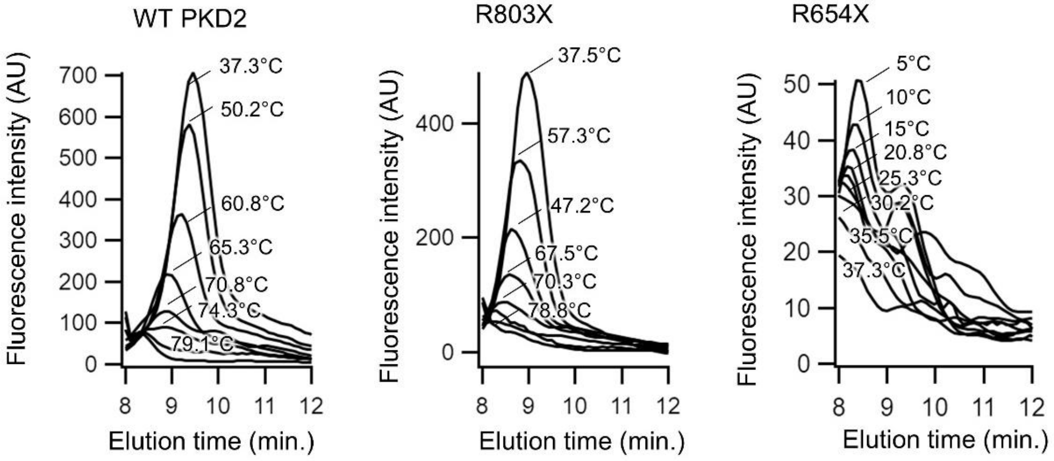

Supplemental Figure 3. PKD2 R654X variant destabilizes channel assembley. Thermal denature profiles (T= 4-80°C) for WT and truncating variant channel. Peak homomeric tetramer elution times were observed 8-10 minutes. A clear thermal transition temperature was less obvious for the R654X varaint.

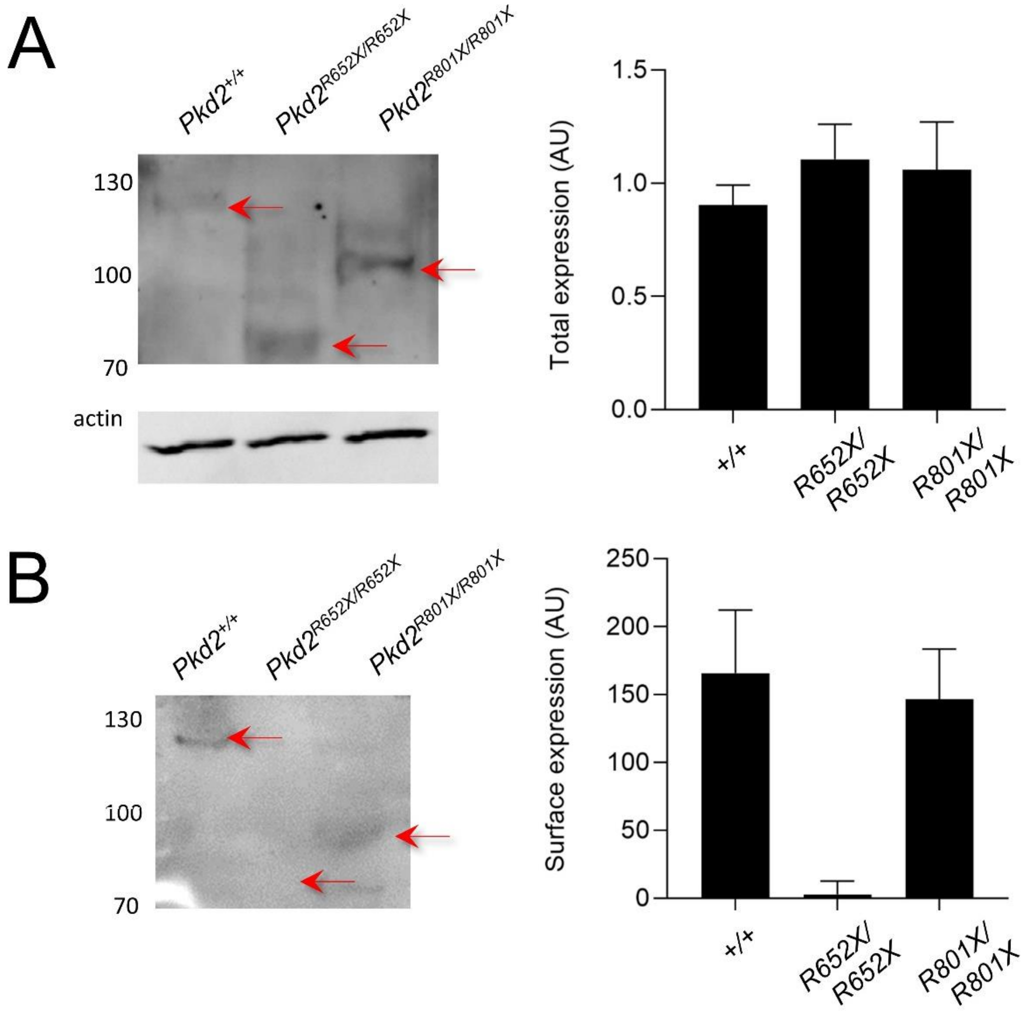

Supplemental Figure 4. Total and surface expression of endenious PKD2 and truncation variants. A) *Left*, western blot of total PKD2 protien harvested from homozygous mice expressing WT (*Pkd2*$^{+/+}$) or truncation varaint (*Pkd2*$^{R652X/R652X}$; *Pkd2*$^{R801X/R801X}$) alleles. *Right*, protien expression as determined anti-PKD2 monoclonal (Sigma Aldrich, WH0005311M1), secondary anti-IgG rabbit (DyLight 488 1:5000) signal intensity normalized to anti-actin protien loading control (MA5-15739, Invitrogen1:5000,). **B**) Biotylation plots detecting surface expression of PKD2 and relatived abundance. N= 4 blots from renal tissue harvested from 4 embryonic mice per genotype.

Channel	Unitary Conductance	$V_{1/2}$ $[Ca^{2+}]_{in}$ = 100 nM	$V_{1/2}$ $[Ca^{2+}]_{in}$ = 30 μM	$\Delta V_{1/2}$
PKD2, WT	97 ± 3 pS	69 mV ± 7	18 mV ± 7	-51 mV
PKD2, R803X	95 ± 3 pS	72 mV ± 8	23 mV ± 8	-49 mV

Supplemental Table 1. Gating properties of PKD2 ADPKD variants. Boltzmann parameters ($V_{1/2}$) and (Z) resulting from fitting the voltage-dependent opening relationship of PKD2 channels reported in Figure 3 and Supplemental Figure 3. Error is equal to S.D.

Genotype	lateral renal cysts	bilateral renal cysts	liver cysts
Pkd2$^{+/R652X}$	25% (3/12)	8% (1/12)	8% (1/12)
Pkd2$^{+/R801X}$	11% (2/14)	0% (0/14)	0% (0/14)
Pkd2$^{+/fl}$ Control	0%, (0/14)	0%, (0/14)	0%, (0/14)
Pkd2$^{+/fl}$ Doxycycline	14%, (2/14)	0%, (0/14)	7%(1/14)
Pkd2$^{R652X/fl}$ Control	28% (2/7)	14% (1/7)	14% (1/7)
Pkd2$^{R652X/fl}$ Doxycycline	*All bilateral*	100%, (5/5)	20% (1/5)
Pkd2$^{R801X/fl}$ Control	28% (2/7)	0% (0/7)	14% (1/7)
Pkd2$^{R801X/fl}$ Doxycycline	*All bilateral*	100%, (7/7)	14%(1/7)

Supplemental Table 2. Histological features of mice expressing ADPKD variants. Incidence of renal (lateral, bilateral) and liver cysts for each murine genotype.

REFERENCES

1. Shen PS, Yang X, DeCaen PG, Liu X, Bulkley D, Clapham DE, et al. The Structure of the Polycystic Kidney Disease Channel PKD2 in Lipid Nanodiscs. *Cell.* 2016;167(3):763-73 e11.
2. Su Q, Hu F, Ge X, Lei J, Yu S, Wang T, et al. Structure of the human PKD1-PKD2 complex. *Science.* 2018;361(6406).
3. Waterhouse AM, Procter JB, Martin DM, Clamp M, and Barton GJ. Jalview Version 2--a multiple sequence alignment editor and analysis workbench. *Bioinformatics.* 2009;25(9):1189-91.
