# Supplementary material for: Molecular pathology and cystogenic propensity of the ADPKD Taiwan founder variant
Source: JCI Insight. 2025 Nov 10;10(21):e191419. doi: 10.1172/jci.insight.191419 (PMC12643504; doi:10.1172/jci.insight.191419)
Supplement: Unedited blot and gel images [file jciinsight-10-191419-s082.pdf]

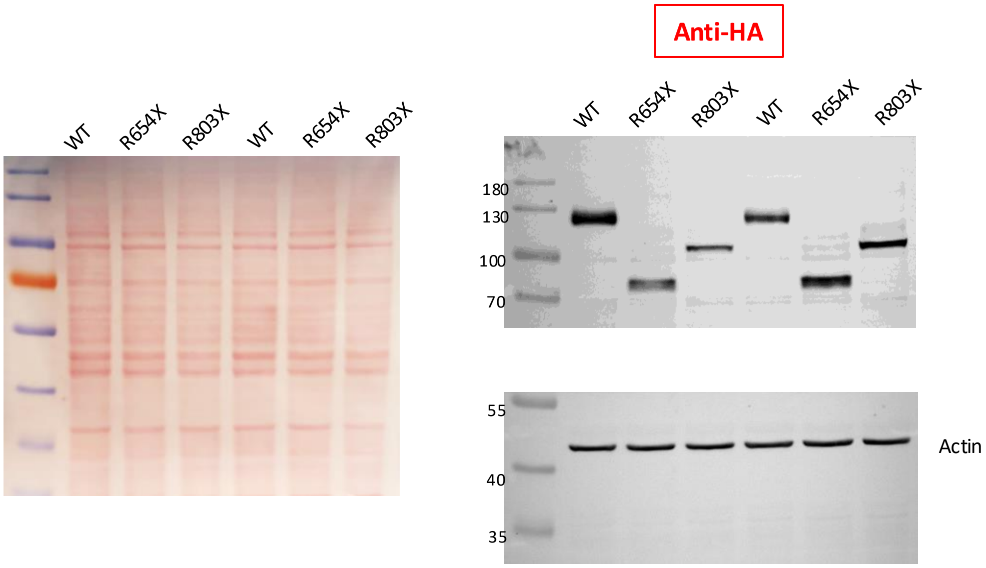

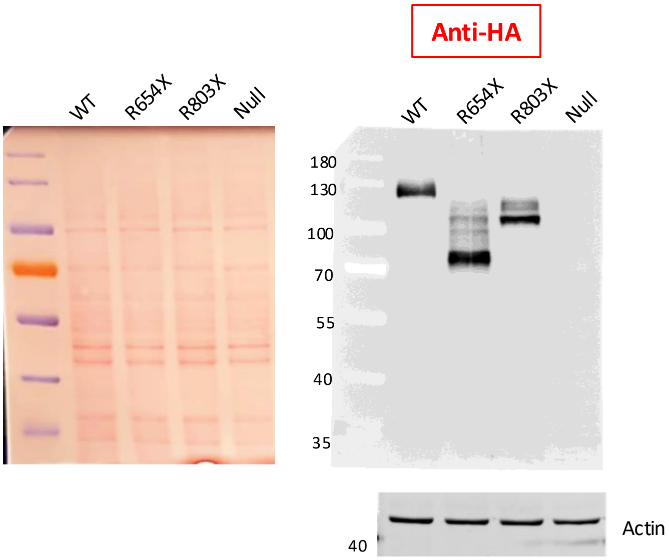

Total blots, relate to Figure 1A

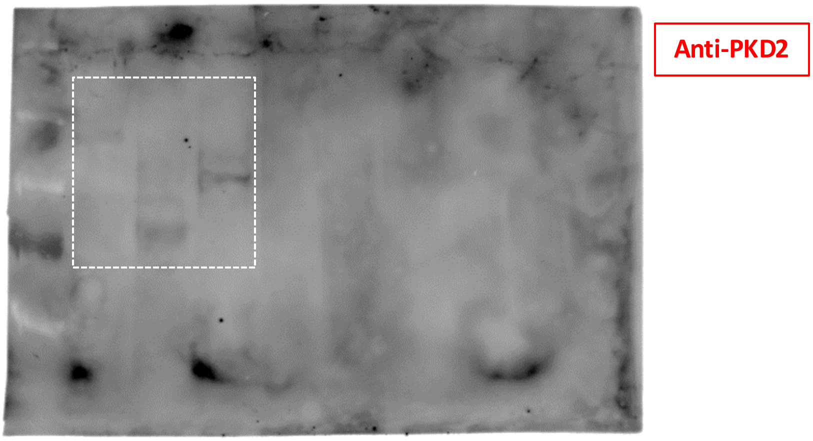

FLUORO_05092025_171757_(1_Alexa Fluor 555)

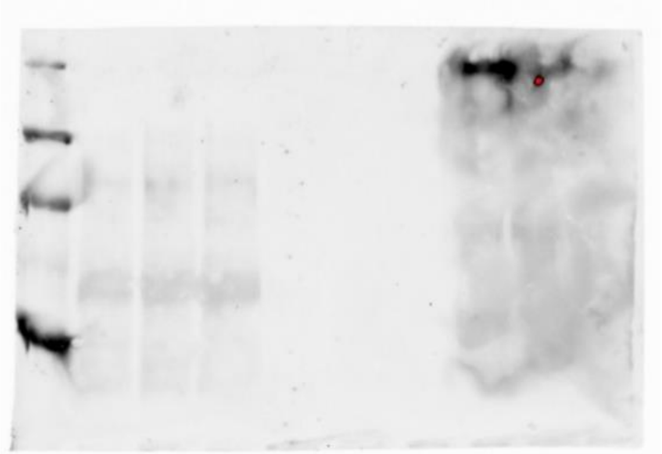

FLUORO_05092025_165926_(3_Alexa Fluor 647)

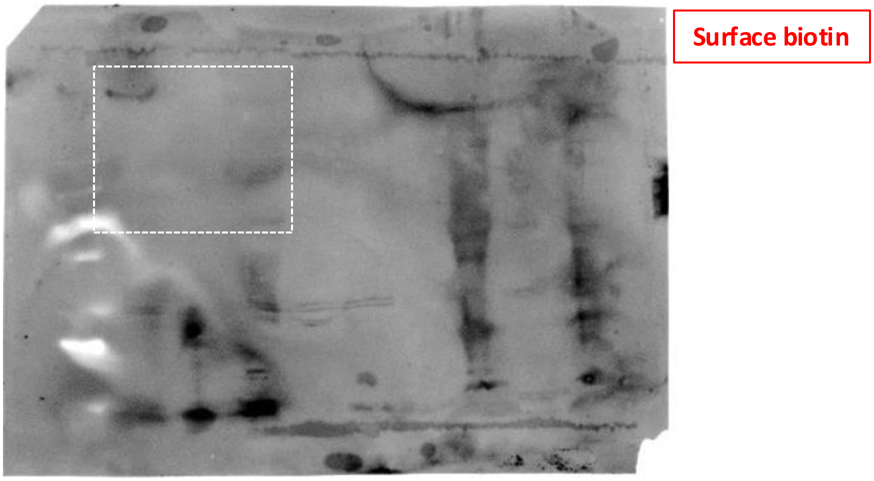

FLUORO_05152025_112826_(1_DyLight 488)

Total blots, relate to supplemental Figure 4A, B
